# Nonintrusive Load Monitoring Based on Advanced Deep Learning and Novel Signature

**DOI:** 10.1155/2017/4216281

**Published:** 2017-10-02

**Authors:** Jihyun Kim, Thi-Thu-Huong Le, Howon Kim

**Affiliations:** ^1^IoT Research Center, PNU, Busan, Republic of Korea; ^2^Pusan National University, Busan, Republic of Korea

## Abstract

Monitoring electricity consumption in the home is an important way to help reduce energy usage. Nonintrusive Load Monitoring (NILM) is existing technique which helps us monitor electricity consumption effectively and costly. NILM is a promising approach to obtain estimates of the electrical power consumption of individual appliances from aggregate measurements of voltage and/or current in the distribution system. Among the previous studies, Hidden Markov Model (HMM) based models have been studied very much. However, increasing appliances, multistate of appliances, and similar power consumption of appliances are three big issues in NILM recently. In this paper, we address these problems through providing our contributions as follows. First, we proposed state-of-the-art energy disaggregation based on Long Short-Term Memory Recurrent Neural Network (LSTM-RNN) model and additional advanced deep learning. Second, we proposed a novel signature to improve classification performance of the proposed model in multistate appliance case. We applied the proposed model on two datasets such as UK-DALE and REDD. Via our experimental results, we have confirmed that our model outperforms the advanced model. Thus, we show that our combination between advanced deep learning and novel signature can be a robust solution to overcome NILM's issues and improve the performance of load identification.

## 1. Introduction

Demand for energy is growing rapidly worldwide, and the demand for electric energy is growing even more rapidly [1]. Electric energy demand will be expected to double between 2010 and 2050 [2]. However, as the global population continues to grow globally and fossil fuels are becoming increasingly depleted, current electricity production methods are not sustainable. The International Electrotechnical Commission (IEC) has stated that the intelligent and economic use of electricity, as the primary energy source, will be the most important factor in solving energy problems [3]. As a result, research is being conducted on ways to efficiently utilize energy in factories, buildings, and homes.

In the field of factories, researches are being carried out on Factory Energy Management Systems (FEMS) for efficient electric energy use. Recently, it has been linked to the Cyber Physical Systems (CPS) of Industry 4.0, and related research will be more active in this area [4]. In the field of buildings, research is underway on Building Energy Management System (BEMS) to reduce unnecessary energy consumption [5]. Depending on the external environment, air conditioners can be appropriately controlled to reduce the electric energy use. Unnecessary energy use can also be reduced through monitoring. In homes, the smart grid will be able to efficiently monitor and manage energy as it becomes increasingly realistic [6].

**Figure 1:**
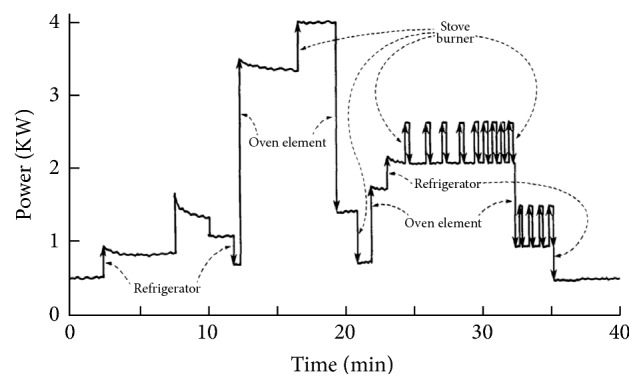
Basic concept of NILM [8].

One approach to increasing the efficiency of domestic electricity use is to inspire positive behavioral change in consumers [7]. This can be achieved by analyzing energy use. Nonintrusive Load Monitoring (NILM) is one alternative to do so. NILM is a process for analyzing changes in the voltage and current going into a house and deducing which appliances are being used in the house along with their individual energy consumption. The initial concept of disaggregating residential power load information was proposed by Hart [8]. He demonstrated how different electrical appliances generate distinct power signatures, including their active power, current, and voltage (Figure 1). He showed how on-off events were sufficient to characterize the use of some appliances. Many researchers have studied this concept and improved the NILM model.

In order to disaggregate a power consumption based on NILM, we have to understand features of appliances. Hart defined three types of appliance model according to the features. Three types are* On/Off*,* Finite State Machine (FSM)*, and* Continuously Variable* [8, 9].

**Figure 2:**
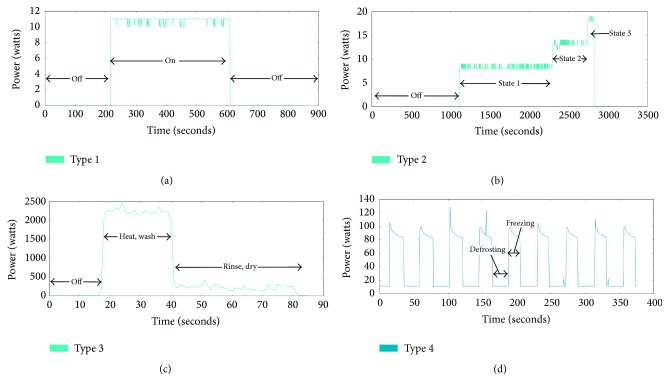
Four types of appliances. (a) Type 1 (On/Off): light; (b) type 2 (FSM): lamp; (c) type 3 (Continuously Variable): washing machine; (d) type 4 (Always On): refrigerator.

The first type* On/Off* has binary states of power such as a light and a toaster.  Figure 2(a) shows the power pattern of light. When it is turned on, the power level is increased from 10 to 12 watts. The appliances of type 1 can be easily classified because of a clear feature. However, if two appliances which have similar power consumption are operating, they could not be distinguished. In this case, a classification model does not know which appliance is generating the current power. Therefore, we need an additional factor to distinguish them.

The second type* FSM* has multiple states of power such as a lamp and a fan. The power pattern of lamp is shown in Figure 2(b). Three operation states are in lamp according to brightness. This kind of appliances can be modeled by FSM. So it could be simple to classify a single appliance of type 2. However, when the multiple power consumption is summed, the classification is become complicated, because it is hard to figure out that the current power is originated in the variable pattern. Therefore, we need to observe the pattern for a period of time in order to classify the appliances.

The third type has a continuous changeable power pattern such as a washing machine and a light dimmer. In Figure 2(c), the power consumption is fluctuated while heating/washing or rinsing/drying a laundry. Strictly speaking, it is a multiple state appliance but it cannot be modeled due to infinite middle states. To classify type 3 appliances, we have to understand the feature and observe the long-range pattern.

Lastly, we define an additional type* Always On*. The appliances of type 4 are always operating except a special case. For example, a refrigerator in Figure 2(d) is operating consistently. The type 4 appliances could have a periodic pattern or a single pattern. According to circumstances, we can reduce the number of appliances when we train the classification model.

The main contributions in this work consist of two points. First one is* a construction state-of-the-art NILM model*. The proposed model is robust against an increase of appliance due to the reduced time complexity. Although we use the low sample rate data, the multistate appliances and the similar power appliances can be classified efficiently. As learning a long-range power pattern result, we can solve the previous problems, which are mentioned in Section 3. We show that the proposed model outperforms via the experimental results with UK-DALE and REDD. Second one is* a discovery of the novel signature*. The proposed signature raises the performance of classification for the multistate appliance. By emphasizing the power variation when we train the model, the variation could be clearly trained. We show the efficiency via the validation experiments in Section 5.3.

This paper is organized the structure as follows. We give problem statement from related works and proposed solutions in Section 2. Section 3 describes two NILM datasets to be used in our experiments. In Section 5, we conduct two main experiments. The first experiment is about validating the expected effects of the novel signature and learning architecture. The second experiment is about measuring the overall performance and comparing with the existing models and state of the art. Final section, we summarize our works and provide the conclusion.

## 2. Related Work

### 2.1. Signature Based Approach

There are two different kinds of signature. The first signature is the steady state. It makes use of steady-state features that are derived under the steady-state operation of the appliances. Hart proposed the concept of NILM and power change method at the same time in his paper [8]. In this method, real power (*P*) and reactive power (*Q*) are used as the input signature. By computing changes of *P* and *Q*, the appliances are classified. Hart demonstrated how different electrical appliances generated distinct power consumption signatures. He showed how on/off events were sufficient to characterize the use of some appliances. The advantage of this method is that we can use a low sampling dataset and it is easy to identify the appliances having high power consumption. However, it is hard to classify the appliances having low power consumption and the multistate appliances which are types 2 to 4 in Figure 2. In addition to that, the appliances having similar power consumption cannot be classified. To improve NILM, other researches have attempted and proposed alternative signature identification techniques. Najmeddine et al. and Figueiredo et al. used the current *I* and the voltage *V* as the signature [11, 12]. They extracted the features such as Root Mean Square (RMS) of the current and peak. These features are well suited for classifying the appliances in the kitchen. However, the multistate appliances cannot be classified as ever. One of the signatures extracted from high sampling rate data is the harmonic of current. There are several researches acquiring the harmonics via Fourier series [13–16]. The appliances of types 1 and 4 are well classified by current harmonics. But it requires the harmonics set of all combinations of the appliances. As increasing the number of appliances, the harmonics for classification increase exponentially. This is not a practical approach and the memory problem could occur. There are fancy approaches for NILM research. Lam et al. proposed the appliance categorization method based on the shape of *V* − *I* trajectory [17]. They categorized the appliances to eight groups with high accuracy. Gupta et al. tried to identify the appliances based on the noise generated from the operation of appliance [18]. However, the noise is easy to be affected by the environment. So it cannot be high efficient signature. In addition, we need to equip the device for measuring the noise. Therefore, it cannot be practical method.

The second signature is the transient state. This signature is less overlapping in comparison with steady-state signatures. However, high sampling rate requirement in order to capture the transients is the major disadvantage. Chang et al. showed that the power consumption while an appliance is turning on can be calculated as a signature [19]. Five years later, they identified the transient physical behavior of power by using wavelet transform [20]. Another way for classification, Leeb et al. proposed the method analyzing the spectral envelope via Short Time Fourier Transform (STFT) [21]. In this way, the method of using transient power as a signature is suitable for classification of types 1 to 3. But it cannot catch type 4. Norford and Leeb showed that the shape of transient data can be a feature [22]. Cole and Albicki used power spikes generated from transition states [23]. This signature is efficient for classification but it is applied to several specific appliances. Like this, the method of using the current transient when the appliances are turning on is suitable for classification of types 1 and 2. But it cannot catch types 3 and 4. Patel et al. sampled the voltage noise extracted from the transient event [24]. They defined three types of noise: on/off transient noise, steady-state line voltage noise, and steady-state continuous noise. However, to use this method, we need to have knowledge about the power flow such as the reactive power, the active power, and phase of voltage relative to current. The method using the voltage noise is suitable for classification of the multistate appliances.

### 2.2. Learning Model Based Approach

**Figure 3:**
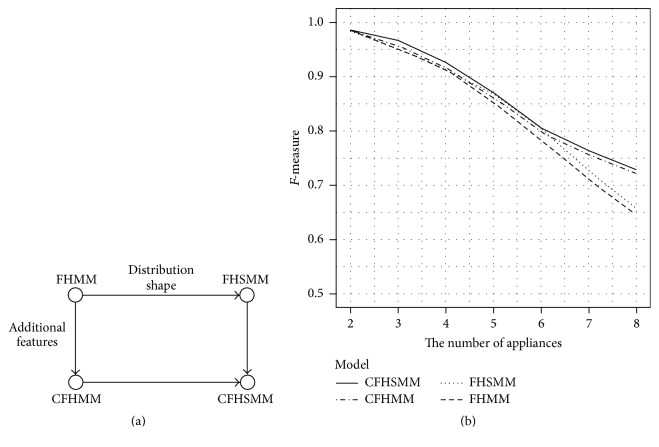
Variants of FHMM and performance results [10]. (a) Relationships between the variant FHMMs; (b) performance comparison.

Some useful temporal graphical models, the variants HMM, are used for NILM. Zoha et al. proposed a solution using FHMM to recognize appliance specific load patterns from the aggregated power measurements [25]. They defined five features that are combinations of the power measurement such as the real power, reactive power, and voltage waveforms. They achieved the *f*-measure of 0.614 for five appliances when they use multistate FHMM. Kolter and Johnson also used FHMM for NILM [26]. They had collected their own dataset called REDD which will be explained in the Section 4. The average accuracy was 47.7%. To improve the researches using FHMM, a number of researchers have extended FHMM. Kim et al. developed probabilistic models of appliance behavior using variants of FHMM [27]. The variants are Factorial Hidden Semi-Markov Model (FHSMM), Conditional FHHH (CFHMM), and Conditional FHSMM (CFHSMM).  Figure 3 shows the relationships between the variant FHMMs and their performance comparison.

Kolter and Jaakkola used a combination of additive FHMM and difference FHMM [28]. For inferencing, they proposed an Additive Fractional Approximate MAP (AFAMAP) algorithm. They achieved the recall of 0.603 for 7 appliances. Parson el al. proposed the variant of the difference HMM and the extended Viterbi algorithm [29]. Zeifman proposed a Viterbi algorithm with Sparse Transitions (VAST) [30]. And he used Markov chain (MC) for NILM.

## 3. Problem Statement and Proposed Solution

### 3.1. Problem Statement

The data for NILM is collected in a timed sequence. Therefore, the models dealing with a sequential data are used as an appliance classification model. The models which are usually used are Factorial Hidden Markov Model (FHMM) based on the Markov process and its variants [32]. However, these models have three problems to classify the appliances. The problems are as follows:

- The first problem is about the time complexity. In case of FHMM, the time complexity is *O*(*Mn*^*M*+1^*T*) where *M* is the number of appliances and *n* is the possible states of hidden unit and *T* is the length of observation sequence. As the number of appliances is increased, the time complexity is increased exponentially as well. This leads to reduction of the classification performance of the model.
- The second problem is about the difficulty of classifying the multistate appliance. The multistate appliance is the appliance which has the multiple state of power consumption. To distinguish them, a long-range pattern should be trained. However, the existing models are based on the first-order Markov process. Therefore, the pattern could not be trained efficiently. In addition, a low sample rate data (e.g., a collected data by 1~6 Hz sample rate) is not proper for classifying the multistate appliances. If we use a high sample rate data for NILM, this problem might be solved due to catching a momentary change of power consumption. But there is a limitation for the real life because a high cost device is needed for collecting the high sample rate data.
- The third problem is a difficulty of classifying the appliances having similar power consumption. If we use only power consumption as an observation of FHMM, the model could not distinguish the similar power appliances.

We surveyed the basic models and the related works of NILM so far. However, there are some problems from the original basics of NILM. In this section, we summarized them into three.

**Problem 1 .**

The researchers have used FHMM and the FHMM variants for NILM [25–27, 29]. As we know, the time complexity of FHMM is *O*(*Mn*^*M*+1^*T*) where *M* is the number of appliances and *n* is the possible states of hidden unit and *T* is the length of observation sequence. Therefore, as increasing the number of appliances, the time complexity also increases exponentially. As a result, the performance of classification model is dropped. We can easily notice that in Figure 3(b).

**Problem 2 .**

The second problem is that FHMM and the FHMM variants are hard to classify type 2 to 4 appliances. A long-term pattern is needed to learn but most models are based on the first-order Markov chain [35]. So these models predict the current state through the previous state. As a result, FHMM and the FHMM variants have a limitation for classifying the multistate appliances. In addition, there is another problem based on the signature perspective. In the signature based approach, we can separate the low sampling rate signature and the high sampling rate signature. The advantage of first signature is that we can easily collect the data from a simple sensor. However, the low sampling rate signature was unsuitable for classifying types 2 to 4 in the most researches [8, 11, 12]. To solve this problem, we need more precise data and high sampling rate signature. By this signature, the researchers classified all types of appliances. However, a specific device should be equipped in order to get the signature. It means that the high cost is required for application of the real life. This is not a good way for popularizing NILM.

**Problem 3 .**

**Figure 4:**
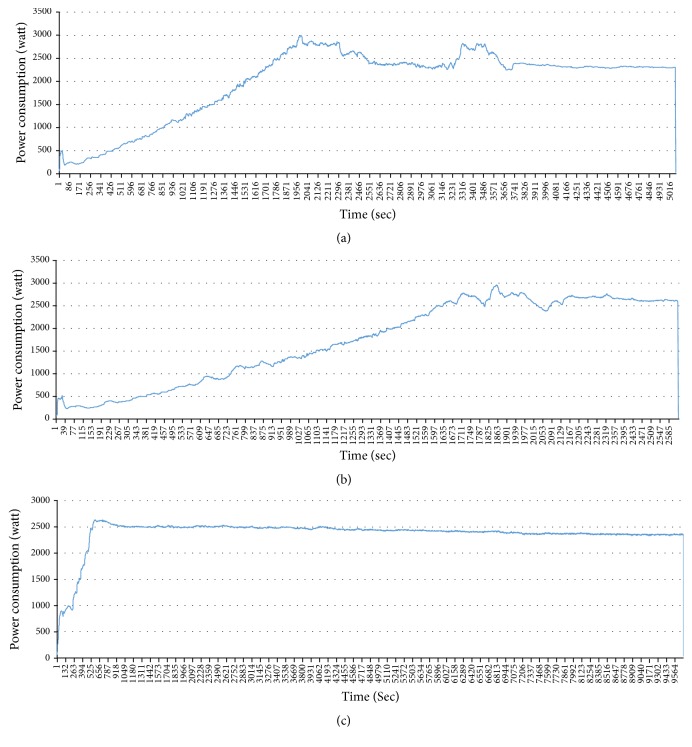
Different durations of air-conditioner: (a) duration: 5016 sec; (b) duration: 2585 sec; (c) duration: 9564 sec.

The last problem is the difficulty of classifying the appliances which have similar power consumption. In the previous models, the observation was only a series of power consumption. HSMM deals with a duration of operation but how can we know the durations for each appliance? The durations are not stable and it is a very tiresome business to extract the durations for each appliance. For example, in Figure 4, we extracted the patterns of air-conditioner from one house. We can notice that the duration is different. It depends on a user's behavior.

Through summarizing the problems, we concluded that the problems from FHMM and the FHMM variants are the fundamental issues from the architecture of FHMM. Therefore, we propose a deep learning based NILM model. Also, we propose the novel low sampling signature in order to apply to the real life. We have three challenges for successful application of deep learning.

*Challenge 1*.* Our model must be robust even though the number of appliances is increased.*

The significant problem of NILM models based on FHMM is that the performance is decreased as increasing the number of appliances. This will be the biggest obstacle for applying NILM to the real life. Therefore, the proposed model should be robust even though the number of appliances is increased.

*Challenge 2. The multistate appliance should be classified.*

The problem when we use a low sample rate data is that it is hard to classify the multistate appliances. This is caused by the fact that various patterns of appliance cannot be reflected efficiently to the main signal (total power consumption) due to a wide sampling gap. Although it is type 1 appliance, the main signal could be a multistate signal because of overlapping signal for each appliance. So we must classify the multistate appliances.

*Challenge 3*.* Although there are the appliances having the similar power consumption, they should be classified.*

If power consumption is the same between two appliances, they would be the same appliances. Except this case, the appliances having the similar power consumption can slightly be different. For the accurate performance, we need to distinguish the similar appliances.

### 3.2. The Proposed Solution

#### 3.2.1. Proposed Novel Signature

Sequence data selected from real life might be power consumption sampled at 1 to 6 Hz. Because of the cost of a sensor, there is a limitation to collecting high sample rate data. To popularize NILM, we have to use low sample rate data from such a sensor. In Section 3, we acknowledge that it is hard to classify multistate appliances (type 2, type 3, and type 4) when using low sample rate data. To solve the problem, we propose a novel signature, which is a key input feature. The main idea of the signature is to separate the original signal, which is low sample rate data (power consumption), using a reflection rate and to subtract one variant power signal from the other variant power signal. We denote the difference as Δ_*p*_ which represents variation of the original signal. The purpose of Δ_*p*_ is to emphasize the variation of multistate appliances by applying the same importance with the original signal to a training model. The result is that performance of the model for an appliance with many variations may be better than when using only the original signal.

To calculate Δ_*p*_, we need to generate the variant power signal. The purpose of using the variant power signal is to reduce noise. An unintended noise can occur and it can have a negative influence on learning the signal pattern. We can regulate noise with the reflection rate. The reflection rate ranges from 0 to 0.99. Power signals that increase or decrease gradually are reflected to the variant power signal, while noise, which occurs in a moment, is not reflected well. This lower reflection rate reduces the negative effect of momentary noise. However, if the reflection rate is too low, the original power pattern may not be reflected. Thus, it is vital to set the reflection rate properly. Through the experiment explained in Section 5.3.1, we empirically found that 0.1 or 0.01 is the proper reflection rate.

**Figure 5:**
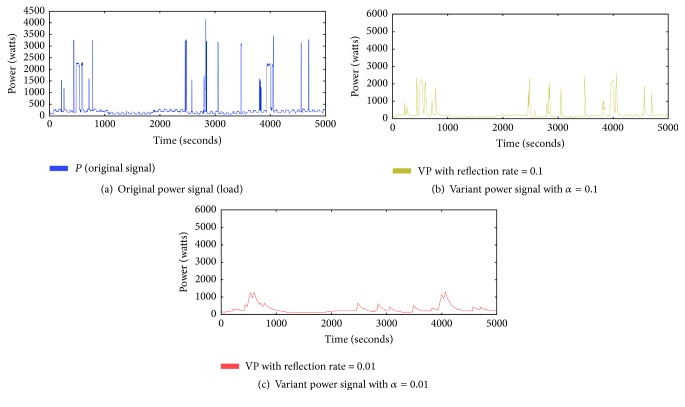
Patterns of the variant power signals.

Algorithm 1 shows how to generate the variant power signal. There are two inputs for the algorithm: *P* is the series of the original signal with the time length *T* and *α* is the reflection rate, which is a ratio for reflecting variation between the original signal and the variant power signal. The output *VP* is the variant power signal generated by the algorithm. In line (1), the variant power signal is initialized to zero. The generation process is given in lines (2) to (5). After the variation at time *i* is calculated in line (3), it is reflected to the variant signal at reflection rate *α* in line (4).  Figure 5 is an example of the variant power signal.  Figure 5(a) is the series of the original signal with the time length 5000.  Figure 5(b) is the variant power signal when the reflection rate is 0.1. Note that the pattern is smoother than the original. This indicates that momentary noise has been reduced while long-range variations remain.  Figure 5(c) is the variant power signal when the reflection rate is 0.01. In this case, the original signal changes are more slowly reflected to the variant power signal. With two different variant power signals, we can calculate the variations in the original signal.

The algorithm for the novel signature is in Algorithm 2. Two variant power signals are the input and the novel signature representing the series of variation is the output. Δ_*p*_ is calculated by subtracting the inputs. In the scale of the original signal, small variations are easily ignored. However, if we give Δ_*p*_ the same weight as the original signal, these small variations become more apparent than before. This effect of the novel signature will be validated in Section 5.3.2.

**Figure 6:**
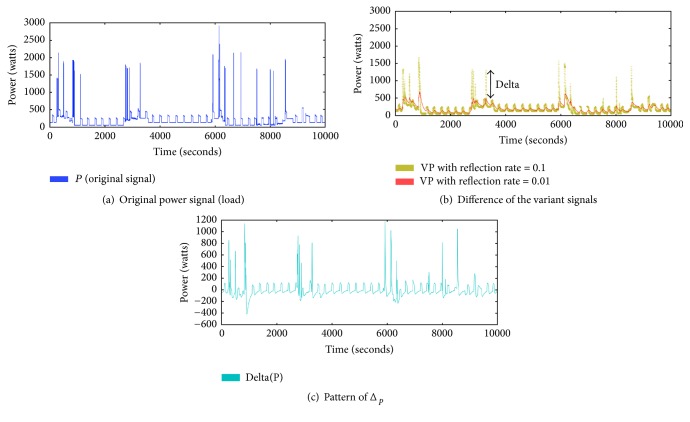
Computing Δ_*p*_ from the variant signals.

Figure 6 represents a computation of Δ_*p*_. By Algorithm 1, the variant power signals (Figure 6(b)) are generated from the original signal (Figure 6(a)). We can see that the patterns of the variant power signal differ according to the reflection rates. The variant power signals in Figure 6(b) can be the inputs for Algorithm 2. Finally, the series of variations, Δ_*p*_, is generated by Algorithm 2.

#### 3.2.2. Learning Architecture

In this section, we introduce how to apply deep learning to NILM. We choose RNN as the learning algorithm. RNN can learn the sequential data such as the power consumption of household. And we apply LSTM to hidden layer in order to prohibit the vanishing gradient problem. We call this model LSTM-RNN. There are two reasons for applying deep learning to NILM. The first reason is that the time complexity was getting higher as the number of appliances was increased in the FHMM variants. The time complexity of FHMM is *O*(*Mn*^*M*+1^*T*) where *M* is the number of appliances and *n* is the possible states of hidden unit and *T* is the length of observation sequence. As we can see, the number of appliances has a close relation with the time complexity. As a result, the performance was dropped as the number of appliances was increased. In deep learning, the major time complexity is originated from backpropagation. Stochastic Gradient Decent (SGD) is usually used for backpropagation [36]. As a result, the time complexity of SGD is *O*(*md*). In our model, the output dimension is the number of appliances. So the time complexity is not increased exponentially in contrast with FHMM. If we use the deep learning based NILM model, the model could be robust relatively. Additionally, deep learning uses the parallel computing via GPU. It means that even though the time complexity is high, GPU can reduce it. This is one of the major reasons why many researchers have been fascinated by deep learning. For the record, the latest GPU, Titan X with Pascal Architecture, has 3584 cores, 12 GB memory, and 11 GFLOP.

**Figure 7:**
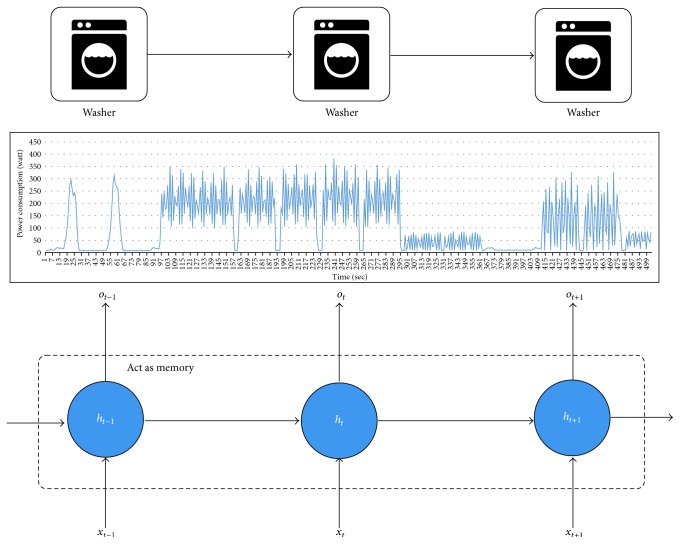
Long-range pattern learning.

The second reason is that the long-term pattern cannot be learned in the FHMM variants (see Figure 7), because they are based on the first-order Markov chain. On the other hand, the hidden layer of LSTM-RNN is a kind of memory. A washer is the representative of the multistate appliance. Although the pattern is long and changeable, it could be learned when we set a length of LSTM-RNN similarly. This feature could classify the multistate appliances and the appliances having the similar power consumption.

We realized that the NILM model based on deep learning has a high possibility of achieving three challenges. One important thing when using LSTM-RNN model is how to choose the suitable factors of this model. Here we discover four factors, which are considered much effective in our model.

*Preprocessing Method*. Generally, *Z*-Score method and Min–Max Scaling are considered as a normalization method for deep learning. In deep learning, a model has better performance when a distribution of input data is close to Gaussian distribution. Min–Max Scaling is the most simple way to normalize the input data. It converts the scale in range from 0 to 1 by using the minimum and maximum value. This method holds the original distribution but it cannot be the Gaussian distribution. *Z*-Score method normalizes the input data by using the mean and standard deviation. This method cannot hold the original distribution when the input distribution is not the Gaussian. Nevertheless, it is important that the mean of input data is close to zero, because if the input is all positive or negative, the weight could be updated in one way. In case of NILM, one of inputs, power consumption, is all positive value. Therefore, we use *Z*-Score as the preprocessing method. We are going to take an experiment about the preprocessing method in Section 5.4.1.

*Weight Initialization Method*. Asymmetry is the most important thing for the weight initialization. We can simply think that if the weights are initialized to zero, the update could be clear. But soon we realize that this method is wrong. There are only bias values in forward propagation. The simplest way to initialize the weights with asymmetry is selecting values from normal distribution or uniform distribution. However, this method has the problem that a variance of output increases with an input dimension. To solve this problem, the variance of output needs to be divided by the square root of input dimension. By this way, Glorot initialization and He initialization are commonly used for the weight initialization. The simple equations of them are as follows:

(1) Wglorot=randomfanin,fanoutfanin,Whe=randomfanin,fanoutfanin/2,

where fan_in_ and fan_out_ are the number of input and output dimensions and *random* is a function of random selection in range from fan_in_ to fan_out_ from normal distribution or uniform distribution. Both equations are similar but He initialization selects the weight values in wide range relatively. In deep neural network like RNN, the weight can easily be shrunk to nothing when the values are close to zero. This leads to vanishing gradient problem. Therefore, we use He initialization due to the wide range value. A related experiment will be conducted in Section 5.4.2.

*Activation Function*. In general, the softmax is used as an activation function for multiclass classification [37]. It can select a one class among all classes. However, in NILM, many appliances could have been operated in the same time. It means that we need to classify the multiple classes. Therefore, we set the boundary with assuming that the appliance is turned on when the output unit value is bigger than the boundary. To do this, we change the softmax to the hyperbolic tangent (tanh) as the activation function. By this way, we can classify the multiple appliances at the same time. The sigmoid can also be the activation function. However, while the output of sigmoid is not zero-centered, the tanh has the zero-centered output. As a result, we could set the static boundary when we use tanh as the activation function.

*Optimizer*. There are three optimizers for SGD. One of them is Adagrad. It decreases a learning rate when a signal is frequent or the weight has a high gradient. In the opposite case, it increases the learning rate. However, this method has a low performance due to decreasing the learning rate fast. RMSProb solves this problem by an exponential moving average. Adam is a combination of RMSProb and Momentum. It is known as the most efficient method for SGD. Therefore we use Adam as an optimizer.

**Figure 8:**
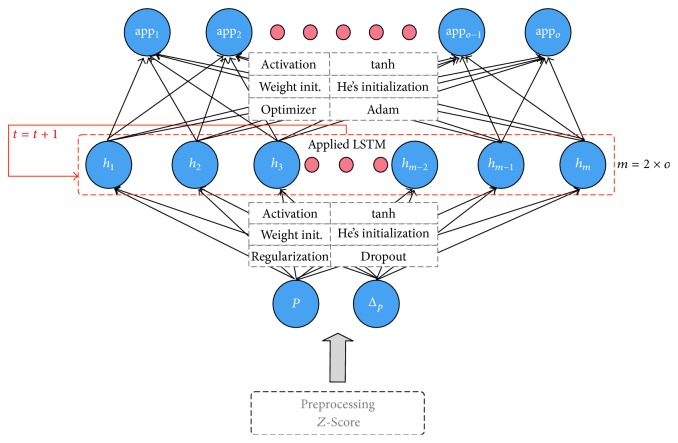
LSTM-RNN for NILM.

The inputs are the original signal (power consumption) and Δ_*p*_. The output unit represents the on/off state of each appliance. By using the novel signature Δ_*p*_, we can expect the better performance for classification of multistate appliances. We apply dropout between the input layer and the hidden layer in order to prevent overfitting. This is the simplest way to regularize the weights and is suitable for deep neural networks like an RNN. We set the number of hidden units to be double the number of output units. A small number of hidden units require more training epochs, whereas a larger number of hidden units require more computation time per epoch. We found that double the number of output units is a proper number for the NILM model through heuristic experiments. Based on these methods, we construct the architecture of an LSTM-RNN for NILM (Figure 8).

## 4. Dataset Description

There are several public datasets for NILM.  Table 1 shows descriptions for them. Among them, we concentrated on two datasets such as REDD and UK-DALE because of proper for NILM. Beside the public dataset, we had collected 6 appliances and aggregate data during 5 months. We are going to use our private dataset as well.

The UK-DALE was released in 2014 at first and it has been updated every year [38]. There are power data for 5 households and each house has the different first measurement date. House 1 includes 3.5-year power data for 52 appliances. The 16 kHz data will not be used in this paper. And houses 1, 2, and 5 include 1-second data but there are no labeled data. Therefore we will not use 1-second data as well. We give an explanation for the 6-second data.  Table 2 shows the detailed description of UK-DALE. House 1 has the large number of appliances. As the number of appliances is increased, a NILM model is hard to distinguish the appliances. But the disadvantage can be overcome because of much data. On the other hand, houses 3 and 4 have the small number of meters. The difference between of them is that a meter of house 4 is shared. For example, “tv_dvd_digibox_lamp” means that the 4 appliances are using the same meter. When the NILM model is trained by a dataset, the shared meter may cause a confusion or make a distinguishable pattern. In Section 5, we can confirm the effect of the shared meter.

The REDD is the first public dataset for NILM [26]. The major purpose of REDD is the standard dataset for benchmarking the NILM algorithms. In REDD, there are AC waveform data with sampling rate of 15 kHz. Therefore, REDD can be used for each approach using the high or low sampling data. It is sampled from six different houses in Massachusetts of the United States. There are three categorized data, low_freq, high_freq, and high_freq_raw. We are going to use low_freq data in this paper. Each house's dataset is composed of the main phases sampled 1 Hz and the individual data of each appliance sampled 3 or 4 Hz.  Table 3 shows the detailed description of REDD. Each house has the appliance in range from eleven to twenty-six. We renamed the same appliances like kitchen_outlets1 and kitchen_outlets2.

The problem of UK-DALE and REDD is the asynchronization between a total load and each appliance data due to the different sample rate. So we have to synthesize each appliance data for generating an output dataset. When we collect the data from the real household, noise can be included in the total load. However, the synthesized data is not affected on the noise. To solve the problem, we collect the data of six appliances for five months. The description of the private data is in Table 4.

## 5. Experiment

### 5.1. Experiment Description

The purpose of the experiment is to satisfy three challenges defined. The challenges are as follows.

*Challenge 1*. Our model must be robust even though the number of appliances is increasing. 

*Challenge 2*. The multistate appliance should be classified as well. 

*Challenge 3*. The similar power consumption in appliances should be classified.

In this section, we take two validation experiments for the proposed signature and learning method and one performance measurement experiment. The first validation experiment consists of two subtests. The first one is a heuristic approach test for the optimized reflection rate. In this test, we compute three different Δ_*p*_ by three different reflection rates. By using Δ_*p*_s, we train the models with the same condition. The second subtest is related to challenge 2. In Section 3.2.1, we expected that the novel signature could classify the multistate appliances having many variations. To validate this, we train two models having {*P*} and {*P*, Δ_*p*_} as inputs for each. And then we analyze the results.

The second validation experiment is related to challenges 1 and 3. This experiment consists of four subtests. In the first subtest, we are going to find an optimized preprocessing method. *Z*-Score normalization and Min–Max Scaling are popular methods for preprocessing. There is no rule which one is proper for a specific situation. So we experiment them for choosing the optimized method. The second subtest is about choice of the weight initialization methods. Among the initialization methods, the Glorot initialization and the He initialization are well known for providing good performance. But it is not revealed that which one is better or not. Therefore, we train two models with only changing the initialization method and compare the result. In the third subtest, we compare the performance as the number of appliances is increased. We could confirm that our model is robust even though the number of appliances is increased. In the last subtest, we synthesize the sampled data of appliances having the similar power consumption. Then, we confirm that the appliances are well classified or not.

The last experiment is about measuring the overall performance. We are going to use the UK-DALE and REDD as the training/test data and measure the performance for each house in the dataset. Also, we take the same experiment by FHMM and compare the result. All models used in the experiment are implemented with Python programming and Theano library.

### 5.2. Performance Metrics

Up to now we have generally assumed that the best way of measuring the performance of a classifier is by its predictive accuracy, that is, the proportion of unseen instances it correctly classifies. However, predictive accuracy on its own is not a reliable indicator of a classifier's effectiveness. As well as the overall predictive accuracy on unseen instances it is often helpful to see a breakdown of the classifier's performance; it is a* confusion matrix* which is proposed by Kohavi and Provost, in 1998 [39].

**Figure 9:**
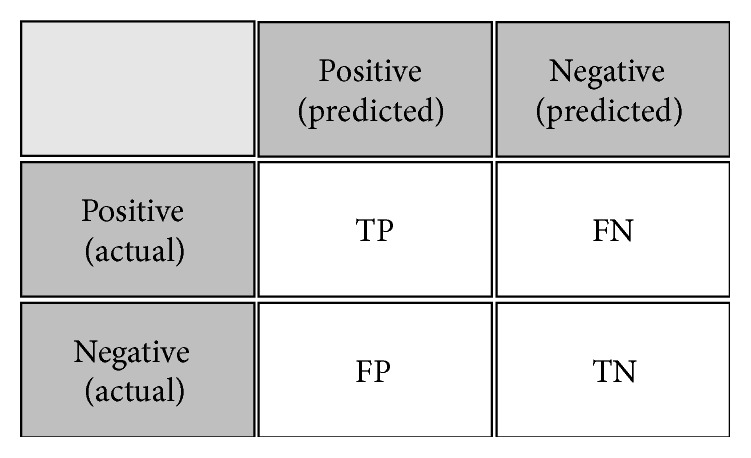
Confusion matrix.

*Confusion matrix* is described in Figure 9, including four categories. True positive (TP) is examples correctly labeled as positive. False positive (FP) refers to negative examples incorrectly labeled as positive. True negative (TN) corresponds to negatives correctly labeled as negative. Finally, false negative (FN) refers to positive examples incorrectly labeled as negative.

We assume that the positive state means that an appliance is turned on. When the appliance is turned off, we regard it as a negative state. Based on the confusion matrix, we can calculate *Recall*, *Precision*, *Accuracy*, and *F*1-*score* for evaluating the NILM model. The formulas to compute the value of them are given by

(2) Recall=TPTP+FN,Precision=TPTP+FP,Accuracy=TP+TNTP+FP+FN+TN,F1-score=2∗Precision∗RecallPrecision+Recall.

*Recall* is a ratio of the number of correct classifications to the total number of actual positive instances. A meaning of that* Recall* is high and the appliances are well classified by the NILM model when the actual instances are positive.* Precision* is a ratio of the number of correct classifications to the total number of predicted positive instances. A meaning of that* Precision* is high and a probability of well classification is high when the NILM model predicts the positive instances.* Accuracy* is a ratio of correct classification to the total test data. *F*1-*score* is the harmonic average of* Recall* and* Precision*.

### 5.3. Proposed Signature Validation Experiment

#### 5.3.1. Optimum Reflection Rate

**Figure 10:**
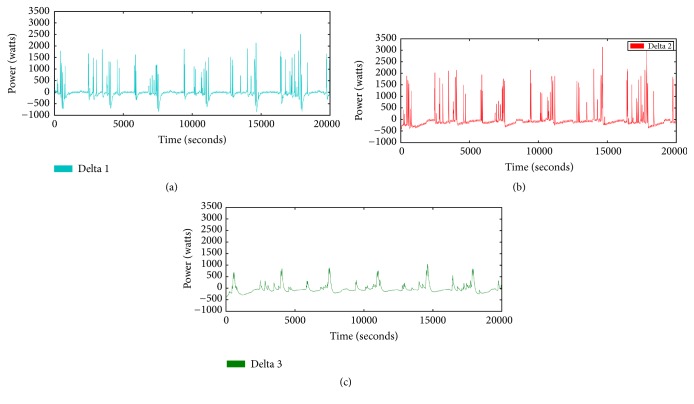
Comparison of Δ_*p*_. (a) Pattern of Δ_*p*_1; (b) pattern of Δ_*p*_2; (c) pattern of Δ_*p*_3.

To find an optimum reflection rate, we set three different reflection rates, 0.1, 0.01, and 0.001, and compute three variant signals. For experiment, we randomly select the 15 appliances from UK-DALE. By using the variant signals, we calculate three Δ_*p*_. Δ_*p*_1 is a result of subtraction of the first signal and the second signal. Δ_*p*_2 is a result of subtraction of the first signal and the third signal. Δ_*p*_3 is a result of subtraction of the second signal and the third signal.  Figure 10 shows the pattern of each Δ_*p*_. Intuitionally, Δ_*p*_2 and Δ_*p*_3 are biased. The values of each Δ_*p*_ are detailed in Table 5. We can notice that the mean of Δ_*p*_1 is close to zero unlike the others. A variance and standard deviation of Δ_*p*_1 are almost in middle of the others. And the scale of range of Δ_*p*_1 and Δ_*p*_2 is similar. We could know which one is proper to NILM by a comparison experiment.

Table 6 shows performance results. All performances are similar except that Δ_*p*_1 is better than the others. Therefore, we will use (0.1, 0.01) as a reflection rate.

#### 5.3.2. Effect of the Novel Signature

In this section, we train the two models having {*P*} and {*P*, Δ_*p*_} as an inputs for each. We extracted 72-day data as a training dataset and 30-day data as a test dataset. Each model was trained 2000 epochs. As we explained in Section 4, the private dataset has six appliances. Actually, these appliances are not operating in the same time. However, in our work, we assumed that multiple appliances could have been operated in the same time. Therefore, 2^6^ = 64 which is the number of combinations of all appliances. We represented the on/off state by the binary representation. And we change the output unit values to one binary string. After that, the string is converted to a decimal number that stands for the combination. A sequence of appliances is {Air-conditioner, Washer, Dehumidifier, Oven, TV, Toaster}. For example, if the washer and toaster are operating, the binary string would be {010001} and the combination number would be 17.

**Figure 11:**
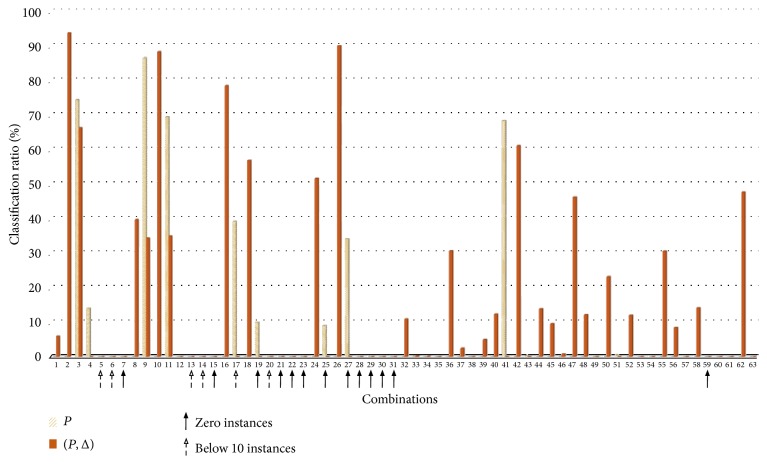
Classification ratio comparison {*P*}, {*P*, Δ_*p*_}.

**Figure 12:**
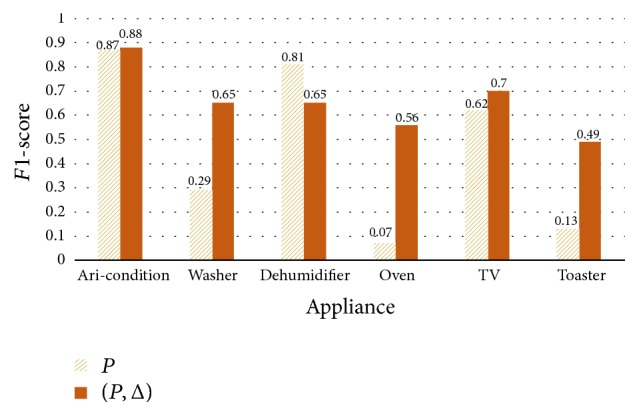
Classification performance of the appliances.

Figure 11 shows the classification ratio comparison between the models. The *x*-axis is a combination and the *y*-axis is a classification ratio. We can easily notice that if we use the proposed signature additionally, the more combinations could be classified. 30 combinations were classified when we use the proposed signature whereas 11 combinations were classified when we use only power consumption (not all combinations are included in the dataset).  Figure 12 shows the performance of each appliance. The second model using {*P*, Δ_*p*_} has the better performance in all appliances except the dehumidifier. Particularly, there are large gaps in the washer, oven, and toaster. The operation time for the oven and toaster is only 1.5 hours and 2.8 hours during 30 days. Even though the sample number is small, the second model has the better performance relatively.

**Figure 13:**
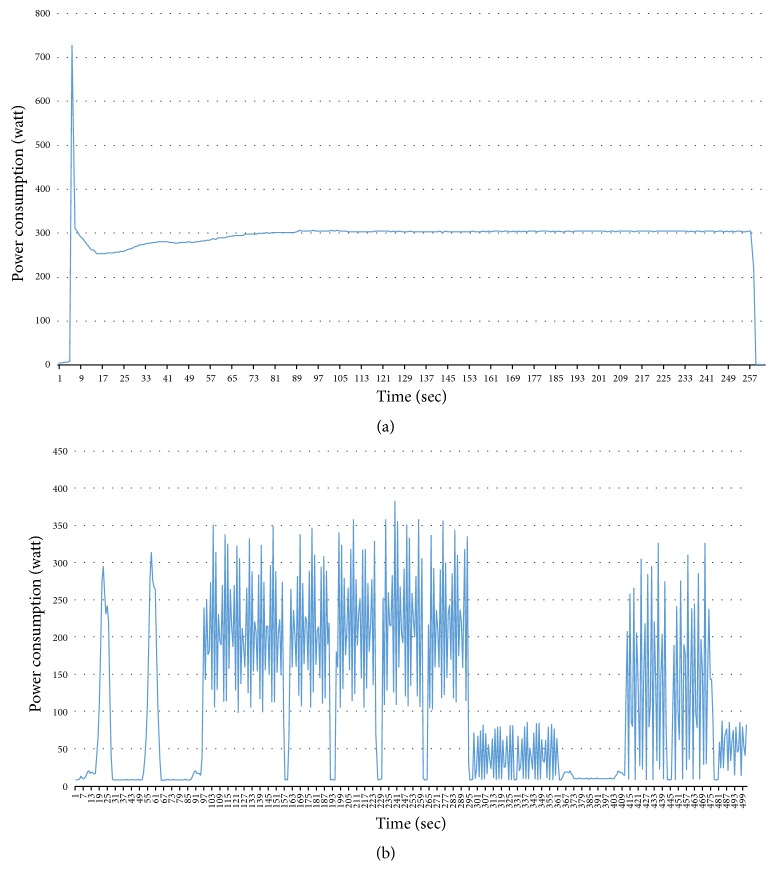
The patterns of dehumidifier and washer: (a) dehumidifier; (b) washer.

However, the dehumidifier performance of first model is higher than that of the second one. We see this performance in Figure 13. The pattern of dehumidifier is simple. A fluctuating Δ_*p*_ could be efficient for learning the pattern but the other case is not. Because the importance of Δ_*p*_ is the same with the power consumption, this kind of simple pattern could be learned slowly. To confirm this, we trained 2000 epochs more for the second model. In Table 7, the performance of dehumidifier is improved from 0.65 to 0.82 whereas the other performances are similar with before. Because the washer has fluctuating Δ_*p*_, the performance of the second model is about double. As a result, the proposed signature could be very efficient factor for classifying the multistate appliances.

### 5.4. Deep Learning Model Validation

#### 5.4.1. Optimum Preprocessing Method

*Z*-Score normalization and Min–Max Scaling are popular methods for preprocessing. However, it has not revealed which one is better. To find an optimum method for NILM, we train the two different models. The datasets for all models are the same. We extracted 23-day data from house 5 of UK-DALE. 17-day data is used as a training dataset and 6-day data is used as a test dataset. Each model trains 1000 epochs.

**Figure 14:**
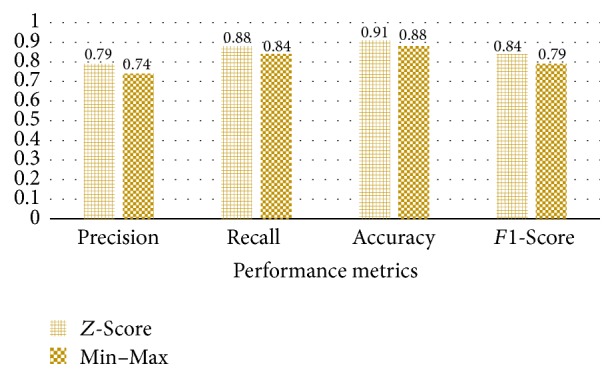
Comparison of the preprocessing methods.

Figure 14 shows the performance results of the models. We can notice that *Z*-Score method has the better performance than Min–Max Scaling. In NILM, one of the inputs is the power consumption. As we know, all power consumption is positive. If all input data are the positive value, all weights could be increased or decreased when the backpropagation is processed. As a result, the dataset for NILM should be standardized. As expected in Section 3.2.2, *Z*-Score method is proper for preprocessing.

#### 5.4.2. Optimum Weight Initialization Method

Among the weight initialization methods introduced, the researchers usually use Glorot initialization and He initialization. But they still have argued about the better method between two methods. The methods use a similar theoretical analysis. They found a good variance for the distribution from which the initial parameters are drawn. This variance is adapted to the activation function used and is derived without explicitly considering the type of the distribution. As such, their theoretical conclusions hold for any type of distribution of the determined variance. To compare the weight initialization methods, we applied the same distribution (uniform distribution) to the methods. We extracted 71-day data from the private dataset. 50-day data are used as a training dataset and 21-day data are used as a test dataset. We train two models during 1000 epochs for each and set a time step to be 500, a loss function to be MSE, and optimization to be Adam.

Table 8 shows the result of experiment. The precision of Glorot initialization is much higher than the precision of He initialization. In case of the recall, He's method is higher than Glorot's method. The model which used Glorot initialization has a high hit rate but a number of classified samples are low. The model which used He initialization has a low hit rate but a number of classified samples are high. The accuracy and *F*1-Score are similar. Before we select the method, we trained the models during 5000 epochs for each.  Table 9 shows the results. After training more than 4000 epochs, the precision of He's method is much improved than the previous result even though the recall is dropped. However, the model which used Glorot initialization is not much improved in all metrics. He initialization was proposed in case the input data is positive. The power consumption is all positive values but Δ_*p*_ is not. We could not convince He initialization. But guessing from the result, we can know that He initialization is efficient for NILM. As a result, we will use He initialization as the weight initialization method.

#### 5.4.3. Performance Measurement with Increasing the Number of Appliances

In this section, we are going to take an experiment for confirming the robustness of proposed model even though the number of appliances is increased. In the experiment, we hold all parameters except the number of appliances. The control parameters are in Table 10. The fifteen appliances for measuring the performance are selected from house 5 of UK-DALE (Table 20).  Table 11 shows the appliances used for each experiment. To observe the performance precisely, we synthesize the data of appliances with adding one appliance to the previous ones.

**Figure 15:**
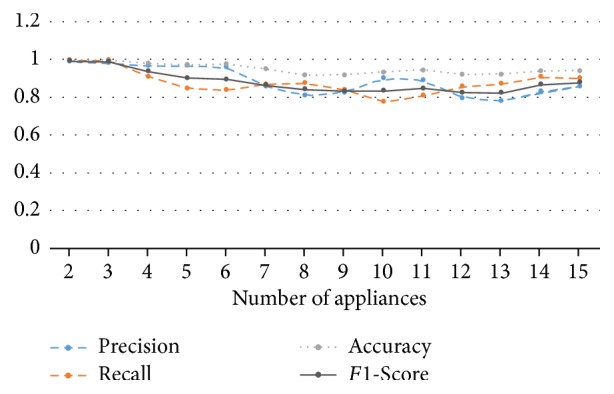
The result of performance measurement model in increasing appliances.

**Figure 16:**
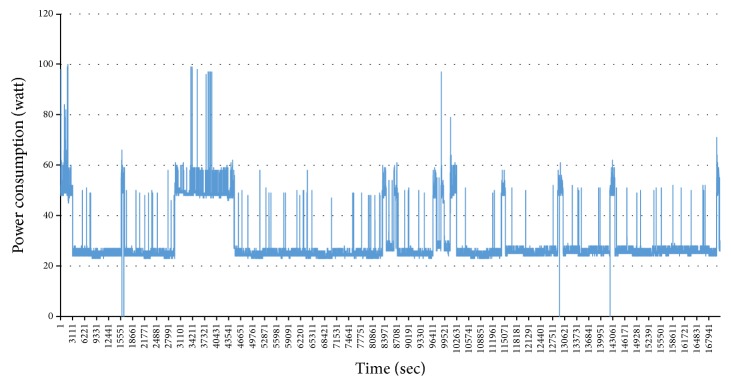
Power pattern of home_theatre_amp.

**Figure 17:**
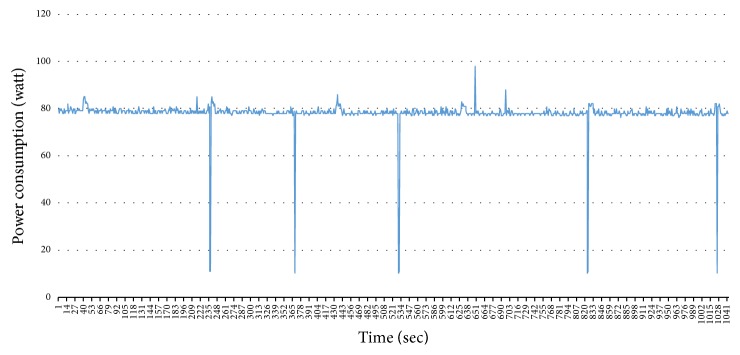
Power pattern of core2_server.

Figure 15 shows the result of experiment. The *x*-axis is the number of appliances. The *F*1-Score representing the overall performance of the model is decreased from 2 to 9. However, after 9 appliances, the *F*1-Score pattern walks up and down in range from 0.8 to 0.9. Due to the high performance, the *F*1-Score is going down in the early phase. However, it is going up and down in the late phase. Therefore, we cannot conclude that the model is influenced by the number of appliances. We compare the two models; those numbers of appliances are 13 and 14 for each. The *F*1-Score of first model is 0.822 but it is increased to 0.866 when the 14 appliances are trained. The added appliance is home_theatre_amp. The total operation time is about 68 hours and we can notice that the appliance has many variations in Figure 16. On the other hand, the *F*1-Score is going down when the number of appliances is from 7 to 8. The added appliance is core2_server. We can notice that the power pattern is monotonous in Figure 17. Therefore, we can think that the major factor affecting on the performance is the data. As a result, we can conclude that the proposed model for NILM is robust against the number of appliances.

#### 5.4.4. Performance Measurement of Appliances Having the Similar Power Consumption

In this section, we are going to take an experiment for confirming that the appliances having a similar power consumption are well classified by the NILM model. If the similar power appliances are operating in a different time, the model could be hard to classify them. However, the power consumption and the operation time are slightly different even though the appliances are the similar power appliance. The power gap would be emphasized by the proposed signature and the operation time could be memorized in the hidden neurons of the proposed model. To validate our assumption, we collected the 11 numbers of the similar power appliances from UK-DALE and synthesized their data in Table 11. And the control parameters are the same with the previous experiment (Table 10).  Table 12 shows the result of performance measurement. We can see that they are well classified even though they are the similar power appliances.

To validate our assumption, we collected the 11 numbers of the similar power appliances and 3 numbers of the multistate appliances having a high power consumption from UK-DALE and synthesized their data in Table 13. And the control parameters are the same with the previous experiment (Table 10). We train two models. The first one is the model using only similar power appliances. In the second model, the multistate appliances are included additionally. As we expected, we can observe that the performance is going down from the first model to the second model in Table 14. Although the performance is decreased, we can realize that.

### 5.5. Performance Comparison with the Existing Models

We could confirm that our proposed signature and model are efficient for NILM through the validation experiments. In this section, we would like to compare the performance with the existing models. We take an experiment for all houses in UK-DALE and REDD. Firstly, we train all houses of UK-DALE and the result will be compared with FHMM. The parameters for UK-DALE experiments are in Table 15.

Table 16 shows the overall performance of UK-DALE houses. The house having the lowest performance is the first house. We proved that our model is affected by the data. There are 53 appliances in the first house. The possibility of being the appliance data decreasing the performance is higher than the other houses. For example, the usage of battery_charger and breadmaker is very rare. This kind of appliance is the major reason for dropping the performance. We compare the performance with FHMM by implementing it. The result is shown in Table 17. We can notice that our model is outperforming FHMM. The performance is almost double in most houses.

Secondly, we train all houses of REDD and the result will be compared with the existing models. The results are good in all houses (see Table 18). We compare our model with three existing models such as FHMM, Additive FHMM, and HieFHMM [28, 32]. Among them, HieFHMM is the state-of-the-art model [34]. Due to few numbers of appliances, the existing models did not test house 2. Similar to UK-DALE result, our model has the highest performance in all houses. In case of house 4, our performance is double compared to that of state of the art. We can confirm this in Table 19.

## 6. Conclusions

In this paper, we considered advanced deep learning is new approach to build state-of-the-art NILM tool. Besides, we proposed a novel signature to overcome multistate appliance issues in NILM. Furthermore, we give three-problem statement and then address them as efficiently. By this way, we perform many experiments for validating the signature and the architecture of the proposed model. Through our experimental results, we realized that our model overcomes the existing problem in energy disaggregation. Via measurement the overall performance, we confirmed that our model outperformed the previous models. In particular, the performance of house 4 in REDD dataset is double result compared to that of state of the art.

In future work, we will focus on a prediction of the power consumption of each appliance, because this problem is different in classification task. We cannot apply our currently model to prediction. Hence, we need to modify our model to generative model. If we can do this, our model could be applied to gas or water disaggregation. In other words, we construct the model which is used for the comprehensive energy management at home.

## Figures and Tables

**Algorithm 1:**
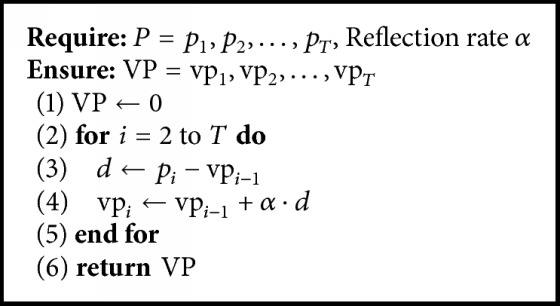
Generation algorithm for the variant power signal.

**Algorithm 2:**
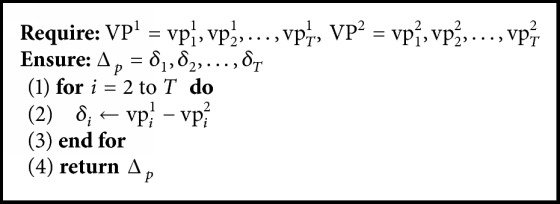
Computing algorithm for the novel signature.

**Table 1:** Public datasets for NILM [31].

Dataset	Number of houses	Duration per house	Appliance sample frequency	Aggregate sample frequency
REDD (2011)	6	3–19 days	3 sec	1 sec, 15 kHz
BLUED (2012)	1	8 days	N/A	12 kHz
Smart (2012)	3	3 months	1 sec	1 sec
Tracebase(2012)	N/A	N/A	1–10 sec	N/A
Sample (2013)	10	7 days	1 min	1 min
HES (2013)	251	1 or 12 months	2 or 10 min	2 or 10 min
AMPds (2013)	1	1 year	1 min	1 min
iAWE (2013)	1	73 days	1 or 6 sec	1 sec
UK-DALE (2016)	5	1–3.5 years	6 sec or 16 kHz	1 or 6 sec, 16 kHz

**Table 2:** The detailed description of UK-DALE.

House	Date of first measurement	Submeters	Appliances
1	2012-11-19	53	Boiler, solar_thermal_pump, laptop, washing_machine, dishwasher, tv, kitchen_lights, htpc, kettle, toaster, fridge, microwave, lcd_office, hifi_office, breadmaker, amp_livingroom, adsl_router, livingroom_s_lamp, soldering_iron, gigE_&_USBhub, hoover, kitchen_dt_lamp, bedroom_ds_lamp, lighting_circuit, iPad_charger, subwoofer_livingroom, livingroom_lamp_tv, and so on

2	2013-02-17	20	Laptop, monitor, speakers, server, router, server_hdd, kettle, rice_cooker, running_machine, laptop2, washing_machine, dish_washer, fridge, microwave, toaster, playstation, modem, cooker

3	2013-02-27	5	Kettle, electric_heater, laptop, projector

4	2013-03-09	6	Tv_dvd_digibox_lamp, kettle_radio, gas_boiler, freezer, washing_machine_microwave_breadmaker

5	2014-06-29	26	Stereo_speakers, desktop, hairdryer, primary_tv, 24_inch_lcd, treadmill, network_attached_storage, server, 24_inch_lcd_bedroom, PS4, steam_iron, nespresso_pixie, atom_pc, toaster, home_theatre_amp, sky_hd_box, kettle, fridge_freezer, oven, electric_hob, dishwasher, microwave, washer_dryer, vacuum_cleaner

**Table 3:** The detailed description of REDD.

House	Submeters	Appliances
1	20	Oven1, oven2, refrigerator, washer_drye1, dishwasher, kitchen_outlets1, kitchen_outlets2, lighting, lighting1 washer_dryer2, microwave, bathroom_gfi, electric_heat, stove, kitchen_outlets3, washer_dryer3kitchen_outlets4, lighting2

2	11	Kitchen_outlets1, lighting, dishwasherstove, microwave, washer_dryer, kitchen_outlets2, refrigerator, disposal

3	22	Outlets_unknown1, kitchen_outlets1outlets_unknown2, lighting 1, microwave, electronics, refrigerator, disposal, dishwasher, furance, lighting2, lighting3, outlets_unknown3, washer_dryer1, washer_dryer2, lighting4, smoke_alarms, lighting5, bathroom_gfi, kitchen_outlets2

4	20	Lighting1, furance, kitchen_outlets1, stove, outlets_unknown, washer_dryer, air_conditioning1, air_conditioning2, miscellaeneous, smoke_alarms, lighting2, kitchen_outlets2, dishwaser, bathroom_gfi1, bathroom_gfi2, lighting3, lighting4, air_conditioning3

5	26	Microwave, lighting1, outlets_unknown1, furance, outlets_unknown2, washer_dryer1, washer_dryer2, subpanel1, subpanel2, electric_heat1, electric_heat2, lighting2, outlets_unknown3, bathroom_gfi, lighting3, refrigerator, lighting4, dishwaser, disposal, electronics, lighting5, kitchen_outlets1, kitchen_outlets2, outdoor_outlets

6	17	Kitchen_outlets1, washer_dryer, stove, electronics,bathroom_gfi, refrigerator, dishwasher, outlets_unknown1, outlets_unknown2, electric_heat, kitchen_outlets2, lighting, air_conditioning1, air_conditioning 2, air_conditioning3

**Table 4:** Private dataset.

House	Date of first measurement	Submeters	Appliances
1	2016-03-11	6	Air-con., dehumidifier, TV, washer, toaster, oven

**Table 5:** Statistic of Δ_*p*_.

Δ_*p*_	Range	Mean	Variance	Standard deviation
Δ_*p*_1	(−841~2510)	1.047	83116.404	288.299
Δ_*p*_2	(−411~3149)	−13.361	140077.047	374.268
Δ_*p*_3	(−366~1040)	−14.358	34845.009	186.668

**Table 6:** Performance measurement with different reflection rates.

Δ_*p*_ (reflection rate)	Precision	Recall	Accuracy	*F*1-Score
Δ_*p*_1 (0.1, 0.01)	0.753	0.843	0.901	0.795
Δ_*p*_2 (0.1, 0.001)	0.714	0.836	0.886	0.771
Δ_*p*_3 (0.01, 0.001)	0.730	0.812	0.888	0.769

**Table 7:** The performance of second model (4000 epochs).

Appliance	*F*1-Score
Air-conditioner	0.89
Washer	0.67
Dehumidifier	0.82
Oven	0.55
TV	0.70
Toaster	0.49

**Table 8:** Performance comparison of initialization methods (1000 epochs).

Metric	He initialization	Glorot initialization
Precision	0.529	0.962
Recall	0.775	0.456
Accuracy	0.934	0.959
*F*1-Score	0.629	0.619

**Table 9:** Performance comparison of initialization methods (5000 epochs).

Metric	He initialization	Glorot initialization
Precision	0.954	0.954
Recall	0.535	0.466
Accuracy	0.964	0.960
*F*1-Score	0.685	0.626

**Table 10:** Control parameters for experiment.

Control parameter	Method
Input	*P*, Δ_*p*_
Num. of training samples	1,500,000
Num. of test samples	500,000
Time step	500
Num. of epochs	1000
Cost function	Mean Squared Error
Optimization	Adam
Preprocessing	*Z*-Score
Weight init.	He initialization
Regularization	Dropout

**Table 11:** A List of the similar power appliances.

Number	Appliance	Power range (watt)
(1)	adsl_router	6~7
(2)	Data_logger_pc	12~13
(3)	hifi_office	12~14
(4)	Livingroom_lamp_tv	11~14
(5)	Livingroom_s_lamp2	7~9
(6)	Modem	9~10
(7)	Office_lamp1	14
(8)	Office_lamp2	9~10
(9)	Server-hdd	10~13
(10)	Speaker	10~11
(11)	Subwoofer_livingroom	15~16

**Table 12:** Result of performance measurement for classification of similar power appliance.

Metric	Similar power appliance based model
Precision	0.955
Recall	0.948
Accuracy	0.957
*F*1-Score	0.951

**Table 13:** A List of the similar power appliances with adding three multistate appliances.

Number	Appliance	Power range (watt)
(1)	adsl_router	6~7
(2)	Data_logger_pc	12~13
(3)	hifi_office	12~14
(4)	Livingroom_lamp_tv	11~14
(5)	Livingroom_s_lamp2	7~9
(6)	Modem	9~10
(7)	Office_lamp1	14
(8)	Office_lamp2	9~10
(9)	Server-hdd	10~13
(10)	Speaker	10~11
(11)	Subwoofer_livingroom	15~16
(12)	Fridge	85~252
(13)	Gas_oven	14~52
(14)	Hoover	500~2021

**Table 14:** Result of performance measurement for classification of similar power appliances with adding three multistate appliances.

Metric	Similar power appliance based model	All appliance based model
Precision	0.955	0.896
Recall	0.948	0.863
Accuracy	0.957	0.911
*F*1-Score	0.951	0.879

**Table 15:** Parameters for UK-DALE experiment.

Parameter	Method
Input	*P*, Δ_*p*_
Time step	500
Num. of epochs	3000
Cost function	Mean Squared Error
Optimization	Adam
Preprocessing	*Z*-Score
Weight init.	He initialization
Regularization	Dropout

**Table 16:** Overall performance of UK-DALE.

House	Precision	Recall	Accuracy	*F*1-Score
1	0.778	0.752	0.917	0.764
2	0.872	0.756	0.905	0.810
3	0.950	0.856	0.981	0.900
4	0.964	0.972	0.969	0.968
5	0.816	0.878	0.914	0.846

**Table 17:** Performance comparison with FHMM (UK-DALE).

House	FHMM	Our model
1	0.304	**0.764**
2	0.423	**0.810**
3	0.495	**0.900**
4	0.651	**0.968**
5	0.424	**0.846**

**Table 18:** Overall performance of REDD.

House	Precision	Recall	Accuracy	*F*1-Score
1	0.942	0.959	0.968	0.950
2	0.961	0.943	0.964	0.952
3	0.829	0.840	0.920	0.835
4	0.846	0.867	0.909	0.856
5	0.930	0.893	0.953	0.911
6	0.898	0.954	0.943	0.925

**Table 19:** Performance comparison with the existing models (REDD).

House	FHMM [33]	Additive FHMM [34]	HieFHMM [34]	Our model
1	0.450	0.749	0.854	**0.950**
3	0.590	0.619	0.834	**0.835**
4	0.430	0.417	0.424	**0.856**
5	0.500	0.795	0.796	**0.911**
6	0.440	0.391	0.820	**0.925**

**Table 20:** Appliances for each experiment.

Number of appliances	Included appliance
2	Stereo_speakers_bedroom, i7_desktop
3	Stereo_speakers_bedroom, i7_desktop, hairdryer
4	Stereo_speakers_bedroom, i7_desktop, hairdryer, primary_tv
5	Stereo_speakers_bedroom, i7_desktop, hairdryer, primary_tv, 24_inch_lcd_bedroom
6	Stereo_speakers_bedroom, i7_desktop, hairdryer, primary_tv, 24_inch_lcd_bedroom, treadmill
7	Stereo_speakers_bedroom, i7_desktop, hairdryer, primary_tv, 24_inch_lcd_bedroom, treadmill, network_attached_storage
8	Stereo_speakers_bedroom, i7_desktop, hairdryer, primary_tv, 24_inch_lcd_bedroom, treadmill, network_attached_storage, core2_server
9	Stereo_speakers_bedroom, i7_desktop, hairdryer, primary_tv, 24_inch_lcd_bedroom, treadmill, network_attached_storage, core2_server, 24_inch_lcd
10	Stereo_speakers_bedroom, i7_desktop, hairdryer, primary_tv, 24_inch_lcd_bedroom, treadmill, network_attached_storage, core2_server, 24_inch_lcd, steam_iron
11	Stereo_speakers_bedroom, i7_desktop, hairdryer, primary_tv, 24_inch_lcd_bedroom, treadmill, network_attached_storage, core2_server, 24_inch_lcd, steam_iron, nespresso_pixie
12	Stereo_speakers_bedroom, i7_desktop, hairdryer, primary_tv, 24_inch_lcd_bedroom, treadmill, network_attached_storage, core2_server, 24_inch_lcd, steam_iron, nespresso_pixie, atom_pc
13	Stereo_speakers_bedroom, i7_desktop, hairdryer, primary_tv, 24_inch_lcd_bedroom, treadmill, network_attached_storage, core2_server, 24_inch_lcd, steam_iron, nespresso_pixie, atom_pc, toaster
14	Stereo_speakers_bedroom, i7_desktop, hairdryer, primary_tv, 24_inch_lcd_bedroom, treadmill, network_attached_storage, core2_server, 24_inch_lcd, steam_iron,nespresso_pixie, atom_pc, toaster, home_theatre_amp
15	Stereo_speakers_bedroom, i7_desktop, hairdryer, primary_tv, 24_inch_lcd_bedroom, treadmill, network_attached_storage, core2_server, 24_inch_lcd, steam_iron, nespresso_pixie, atom_pc, toaster, home_theatre_amp, sky_hd_box

## Abbreviations

NILM: Nonintrusive Load Monitoring
FHMM: Factorial Hidden Markov Model
GPU: Graphics Processing Unit
UK-DALE: UK Domestic Appliance Level Electricity
REDD: Reference Energy Disaggregation Data
HMM: Hidden Markov Model
FSM: Finite State Machine
MP: Markov process
EM: Expectation Maximization
HSMM: Hidden Semi-Markov Model
DHMM: Difference Hidden Markov Model
DBN: Deep Belief Network
RBM: Restricted Boltzmann Machine
AI: Artificial Intelligence
AAT: Automatic Alternative Text
ANN: Artificial Neural Network
tanh: Hyperbolic tangent
MSE: Mean Squared Error
CE: Cross Entropy
GD: Gradient Descent
RNN: Recurrent Neural Network
BPTT: Backpropagation through Time
RTRL: Real Time Recurrent Learning
FFNN: Feed-Forward Neural Network
LSTM: Long Short-Term Memory
PCA: Principal Component Analysis
ReLU: Rectifier Linear Unit
SGD: Stochastic Gradient Decent
RMS: Root Mean Square
STFT: Short Time Fourier Transform
FHSMM: Factorial Hidden Semi-Markov Model
CFHMM: Conditional Factorial Hidden Markov Model
CFHSMM: Conditional Factorial Hidden Semi-Markov Model
AFAMAP: Additive Fractional Approximate MAP
VAST: Viterbi Algorithm with Sparse Transitions
MC: Markov chain
CT: Current Transformer
BLUED: Building Level Fully Labeled Data Set for Electricity Disaggregation
CMU: Carnegie Mellon University
UMASS: University of Massachusetts
HES: Household Electricity Use Study
AMPds: Almanac of Minutely Power Dataset
IIIT: Indraprastha Institute of Information Technology
iAWE: International Associations for Wind Engineering
TP: True positive
FP: False positive
TN: True negative
FN: False negative
HieFHMM: Hierarchical Factorial Hidden Markov Model
HW: Hamming weight
WISA: International Workshop on Information Security Applications
MBC: Mask based Block Comb
NCC: Normalized Cross Correlation.
