# Corrigendum to “Nonintrusive Load Monitoring Based on Advanced Deep Learning and Novel Signature”

**DOI:** 10.1155/2018/7080564

**Published:** 2018-04-30

**Authors:** Jihyun Kim, Thi-Thu-Huong Le, Howon Kim

**Affiliations:** ^1^IoT Research Center, PNU, Busan, Republic of Korea; ^2^Pusan National University, Busan, Republic of Korea

In the article titled “Nonintrusive Load Monitoring Based on Advanced Deep Learning and Novel Signature” [1], an acknowledgment should be added as follows.

This material is based upon work supported by the Ministry of Trade, Industry & Energy (MOTIE, Korea) under Industrial Technology Innovation Program (10073236).
